# The Essential DNA Damage Response Complex MRN Is Dispensable for the Survival and Function of Purkinje Neurons

**DOI:** 10.3389/fnagi.2021.786199

**Published:** 2022-01-28

**Authors:** Mingmei Ding, Xiaobing Qing, Guangyu Zhang, Carolin Baade-Büttner, Ralph Gruber, Haizhen Lu, David O. Ferguson, Christian Geis, Zhao-Qi Wang, Zhong-Wei Zhou

**Affiliations:** ^1^School of Medicine, Shenzhen Campus of Sun Yat-sen University, Shenzhen, China; ^2^Leibniz Institute on Aging – Fritz Lipmann Institute (FLI), Jena, Germany; ^3^Section of Translational Neuroimmunology, Department of Neurology, Jena University Hospital, Jena, Germany; ^4^Department of Pathology and Resident Training Base, National Cancer Center/Cancer Hospital, Chinese Academy of Medical Sciences and Peking Union Medical College, Beijing, China; ^5^Department of Pathology and Comprehensive Cancer Center, University of Michigan Medical School, Ann Arbor, MI, United States; ^6^Faculty of Biological Sciences, Friedrich-Schiller-University of Jena, Jena, Germany

**Keywords:** NBS1, MRE11, Purkinje neurons, cerebella, neurodegeneration

## Abstract

MRE11, RAD50, and NBS1 form the MRN complex in response to DNA damage to activate ATM, a gene responsible for Ataxia-Telangiectasia (A-T). Loss of any components of the MRN complex compromises cell life. Mutations in *MRE11*, *RAD50*, and *NBS1* cause human genomic instability syndromes Ataxia-Telangiectasia-like disorder (A-TLD), NBS-like disorder (NBSLD), and Nijmegen Breakage Syndrome (NBS), respectively. Among other pathologies, neuronal deficits, including microcephaly, intellectual disabilities, and progressive cerebellar degeneration, are common in these disorders. *Nbs1* deletion in neural stem cells of mouse models resulted in cerebellar atrophy and ataxia, mimicking the A-T syndrome suggesting an etiological function of MRN-mediated DDR in neuronal homeostasis and neuropathology. Here we show that deletion of *Nbs1* or *Mre11* specifically in Purkinje neurons of mouse models (*Nbs1*-PCΔ and *Mre11*-PCΔ, respectively) is compatible with cerebellar development. Deleting *Nbs1* in Purkinje cells disrupts the cellular localization pattern of MRE11 or RAD50 without inducing apparent DNA damage, albeit impaired DNA damage response (judged by 53BP1 focus formation) to ionizing radiation (IR). However, neither survival nor morphology of Purkinje cells and thus locomotor capabilities is affected by *Nbs1* deletion under physiological conditions. Similarly, deletion of *Mre11* in Purkinje cells does not affect the numbers or morphology of Purkinje cells and causes no accumulation of DNA damage. *Mre11*-deleted Purkinje cells have regular intrinsic neuronal activity. Taken together, these data indicate that the MRN complex is not essential for the survival and functionality of postmitotic neurons such as Purkinje cells. Thus, cerebellar deficits in MRN defect-related disorders and mouse models are unlikely to be a direct consequence of loss of these factors compromising DDR in postmitotic neurons such as Purkinje cells.

## Introduction

The integrity of the genome, the carrier of genetic information, is frequently threatened by extrinsic and intrinsic factors that can cause DNA base damages, mismatches, strand breaks, and other types of DNA damage (Chatterjee and Walker, 2017). To maintain the genome stability and transmit the genetic information faithfully to progenies, cells have evolved a complex system, namely DNA damage response (DDR) pathways, to activate cell cycle checkpoints, alter the transcription process, repair damaged DNA, or induce senescence and even apoptosis if the damage is too severe to be repaired (Ciccia and Elledge, 2010; Lord and Ashworth, 2012; Ghosal and Chen, 2013; Carusillo and Mussolino, 2020). The MRN complex, consisting of MRE11, RAD50, and NBS1 (also known as Nibrin, p95), is one of the most critical DDR complexes that play a crucial role in activating the protein kinase ATM (ataxia-telangiectasia mutated), a member of the PIKK (PI3K-like protein kinase) and an apical activator of DNA double-strand breaks (DSBs) (Paull, 2015; Lu et al., 2021). MRN also functions to trigger activation of another vital DDR-related PIKK member ATR (Ataxia Telangiectasia and Rad3-related) in response to replication fork stalling or DNA single-strand breaks (SSBs) depending on cell cycle status and physiological conditions (Syed and Tainer, 2018; Lu et al., 2021). Activated ATM and ATR phosphorylate hundreds of their substrates, thereby regulating downstream processes of DDR (Blackford and Jackson, 2017). In addition, the MRN complex, through processing broken DNA ends, directly participates in the repair of DSBs *via* non-homologous end joining (NHEJ) or homologous recombination (HR) (Syed and Tainer, 2018). Due to the critical roles in handling most toxic DSBs or damages from replication stress, all components of the MRN complex, similar to ATR, CHK1, TopBP1, BRCA1/2, and other key DDR molecules, are essential for the survival of proliferating cells.

Mutations in genes encoding key DDR molecules often cause human genomic instability syndromes. For example, mutations of ATM lead to Ataxia-Telangiectasia (A-T), of ATR lead to Seckel Syndrome (ATR-SS), of components of the MRN complex cause Ataxia-Telangiectasia-like disorder (A-TLD, mutations in *MRE11*), Nijmegen breakage syndrome-like disorder (NBSLD, mutations in *RAD50*) and Nijmegen breakage syndrome (NBS, mutations in *NBS1*), respectively. Interestingly, neurological deficits are common among these syndromes besides other pathological symptoms such as immunodeficiency, cancer-prone, and radiation sensitivity (McKinnon, 2017). Microcephaly is presented in ATR-SS, NBSLD, and NBS, whereas ataxia is a shared feature of A-T and A-TLD patients (Lavin, 2008; Stracker and Petrini, 2011; McKinnon, 2017). A compromise of neurogenesis generally causes microcephaly during brain development, and ataxia is regarded as a consequence of cerebellar defects and the malfunction of Purkinje cells (McKinnon, 2017).

Neural development, which includes proliferation and differentiation of neural stem cells (or neuroprogenitors), migration of newborn neurons, neuritogenesis/arborization, and synapsis formation, is strictly regulated by different signaling pathways (Stiles and Jernigan, 2010). The rapid proliferation of embryonic neuroprogenitors generates a high level of DNA lesions raised from replication forks, in which a robust DDR machinery is therefore commanded (McConnell et al., 2017; Wang et al., 2020). The defective DDR causes an accumulation of DNA damage and subsequently ceases proliferation or eventually promotes apoptosis. Therefore neuroprogenitors are hypersensitive to malfunction DDR, which often links DDR defects and neurodevelopmental disorders (McKinnon, 2017). To decipher the neurodevelopmental defect of genomic instability syndromes, *Nbs1* was specifically deleted in neural stem cells of the mouse central nervous system (CNS) (*Nbs1*-CNSΔ) in which the neuroprogenitor population was severely affected (Frappart et al., 2005). A blockage of proliferation and a trigger of ATM-P53 mediated cell death of cerebellar neuroprogenitors cause microcephaly, cerebellar atrophy, and ataxia in *Nbs1*-CNSΔ mice (Frappart et al., 2005; Assaf et al., 2008; Dar et al., 2011; Galron et al., 2011; Zhou et al., 2012). Severe microcephaly and cerebellar atrophy were also described in *Atr-*CNSΔ mice (Lee et al., 2012; Zhou et al., 2012). Interestingly, whereas deletion of *Nbs1* in the CNS affects only the survival of proliferating progenitors, *Atr* knockout causes cell death in both proliferating progenitors and postmitotic neurons of the cortex, leading to much stronger microcephaly in *Atr*-CNSΔ mice than *Nbs1*-CNSΔ mice (Zhou et al., 2012). These studies indicate that defects of the essential DDR molecules affect the fate of neuroprogenitors and, therefore, the development of the cortex and cerebellum.

Ataxia is commonly caused by the dysfunction or degeneration of Purkinje cells (Hoxha et al., 2018). A-T patients display abnormalities in morphology and localization of Purkinje cells (Vinters et al., 1985; Borghesani et al., 2000; McKinnon, 2017). While A-T patients exhibit cerebellar atrophy and ataxia, *Atm* knockout mouse models do not recapitulate these phenotypes. Additionally, although NBS1 and MRE11 function together mainly to activate ATM and repair DSBs, only A-TLD, but not NBS, patients show ataxia, suggesting a different yet unknown function of each component of the MRN complex controlling cerebellar development or the function of Purkinje cells. Intriguingly, the *Nbs1*-CNSΔ mouse model mimics neuropathological symptoms of A-T patients, namely cerebellar atrophy and ataxia, which can be corrected mainly by knocking out *p53* (Frappart et al., 2005; Zhou et al., 2012). These mouse model studies raise an interesting question as to whether these DDR molecules have direct and different roles in developing the cerebellum or maintaining Purkinje cell function.

In the present study, we deleted *Nbs1* or *Mre11* specifically in Purkinje cells to generate *Nbs1*-PCΔ and *Mre11*-PCΔ mice, respectively. Although deletion of *Nbs1* leads to dislocation of other components of the MRN complex and impairment of DDR in Purkinje cells, mutant mice show a typical architecture of the cerebellum and possess normal locomotor capabilities. Similarly, neither cellular density nor neuronal activity is affected after deletion of *Mre11* in Purkinje cells, highlighting that NBS1 or MRE11 is not essential for the survival and functionality of Purkinje cells.

## Materials and Methods

### Mice and Genotyping

*Nbs1-*PCΔ or *Mre11*-PCΔ mice with a specific deletion of *Nbs1* or *Mre11* in Purkinje cells were generated by crossing *Nbs1*^flox/flox^ (Frappart et al., 2005) or *Mre11*^flox/flox^ (Buis et al., 2008) mice with Pcp2-Cre transgenic mice (Kirtay et al., 2021). *Nbs1-*CNSΔ; P53-/- mice were generated previously described (Frappart et al., 2005). All the mice were maintained in the mouse facility of Fritz Lipmann-Institute (FLI, Jena, Germany). Mice were fed *ad libitum* with standard laboratory chow and water in ventilated cages under a 12 h light/dark cycle. All animal breeding and experiments were conducted according to the animal welfare licenses approved by the Thüringer Landesverwaltungsamt (animal license 03-042/16) and license issued by SYSU Institutional Animal Care and Use Committee (SYSU-IACUC-2019-B1028). The genotypes of mice were confirmed by PCR using primers, as follows. For *Nbs1*: exon6 (cagggcgacatgaaagaaaac), Intron5F (ataagacagtcaccactgcg) and LoxPtestR (aatacagtgactcctggagg); for *Mre11:* Loxup1 (tacaaaaggttgaaaatttgagaagc) and LoxD1: (taggtagctacaaacatatatctgc); for *Cre*: Cre1 (cggtcgat gcaacgagtgatg), Cre2 (ccagagacggaaatccatcgc), B2-1 (caccgga gaatgggaagccgaa) and B2-2 (tccacacagatggagcgtccag).

### Rotarod Performance Test

Before the testing period, the mice were trained for 1 day on the Rotarod apparatus (Ugo Basile, Italy) at a constant speed (5 rpm) for a maximum of 5 min to let them adapt to the testing environment. During the testing period, each mouse was placed on the Rotarod at an accelerated speed (10 rpm/min), from 5 to 50 rpm for a maximum of 300 s. When the mice fell, they were removed from the apparatus and placed back into their cage to recover at least 30 min before the subsequent trial. All the mice received three trials per day for four to five consecutive days. Latency to fall served as an indicator of motor coordination.

### Ionizing Radiation

Mice were irradiated with 15 Gy of ionizing radiation using ^137^Cs γ–irradiation source (Gammacell 40, Nordion, Ottawa, ON, Canada). Mice were sacrificed, and brain samples were collected 30 min after irradiation.

### Histological and Immunofluorescence Analyses

Mouse brains were isolated from indicated age of *Nbs1*-PCΔ or *Mre11*-PCΔ mice and fixed in 4% paraformaldehyde (PFA) at 4°C for 24 h. For the paraffin sections, the fixed brains were embedded and cut in 4-μm thickness for H&E staining. For the preparation of cryosections, the brains were fixed in 4% PFA overnight and cryoprotected with 30% sucrose in PBS at 4°C for 1–2 days. The brains were then embedded in a cytomatrix (Neg-50, Richard- Allan Scientific, Kalamazoo, MI, United States) and snap-frozen in liquid nitrogen. The sections were prepared at a thickness of 10∼14 μm and stored at 80°C before use. Immunofluorescence staining of cryosections was performed as described previously (Zhou et al., 2013).

The following antibodies and dilutions were used for immunofluorescence staining: rabbit anti-MRE11 (1:400, NB100-142, Novus Biologicals, Littleton, CO, United States), rabbit anti-calbindin D28K (D28K, 1:1,000, Santa Cruz, #7691), mouse anti-phospho-H2AX (ser139) (γ-H2AX) (1:100, 05-636, Upstate, NY, United States) and rabbit anti-53BP1 (1:200, NB100-304, Novus Biologicals, Littleton, CO, United States). anti-mouse Cy3 (1:200, Sigma, #C2181-1Ml), anti-rabbit Cy3 (1:200, Sigma, #C2306-1Ml), anti-rabbit Cy2 (1:200, Jackson Immuno Research, #711-225-152), Alexa Fluor 488 donkey anti-Mouse IgG (H + L) (1:200, Invitrogen, #A21202). After staining, DNA was counterstained with DAPI. Slides were mounted with ProLongTM Gold Antifade Mountant (Thermo Fisher Scientific). Images were acquired with Zeiss Axio Imager-ApoTome Axiovert 200 ApoTome or a confocal microscope (LSM510, Zeiss, Jena, Germany).

### TUNEL Assay in Brain Sections

TUNEL assay was performed as previously described (Kirtay et al., 2021). After the Tunel staining, sections were incubated by the first antibody rabbit anti-Calbindin (1:200, Swant) at 4°C overnight. After washing with PBS 3 times, sections were incubated by secondary antibody (1:200, Sigma) together with DAPI (1:5,000, Invitrogen) at room temperature for 1.5 h. Images were captured by the ApoTome microscope (Zeiss Jena, Germany) under 40 × objectives.

### Quantification of Purkinje Cells

The whole cerebellum section with H&E staining or calbindin-D28K staining was imaged with a virtual microscope (BX61VS, Olympus, Tokyo, Japan). The total number of Purkinje cells in indicated or each individual cerebellar lobule was quantified by ImageJ software and validated manually.

### Cerebellar Electrophysiology

Electrophysiological recording of Purkinje cells was performed as described previously (Kirtay et al., 2021). Briefly, the brains of 18–20-month-old mice were removed in ice-cold protective cutting artificial cerebrospinal fluid (aCSF1) containing: 95 mM of *N*-Methyl-D-glucamine, 30 mM of NaHCO_3_, 20 mM of HEPES, 25 mM of glucose, 2.5 mM of KCl, 1.25 mM of NaH_2_PO_4_, 2 mM of thiourea, 5 mM of sodium ascorbate, 3.0 mM of sodium pyruvate, 10 mM of MgSO_4_, 0.5 mM of CaCl_2_, 12 mM of *N*-acetylcysteine, adjusted to pH 7.3 and an osmolarity of 300–310 mOsmol, saturated with 95% O_2_/5% CO_2_. 350 μm thick sagittal slices were made from the cerebellum with a vibratome (VT1200S; Leica, Wezlar, Germany). Slices were placed in an incubation beaker with aCSF1 at 34°C for 10–15 min, then transferred into another incubation beaker at room temperature with aCSF2 (containing: 125 mM of NaCl, 25 mM of NaHCO_3_, 25 mM of glucose, 2.5 mM of KCl, 1.25 mM of NaH_2_PO_4_, 1 mM of MgCl_2_, 2 mM of CaCl_2_, 2 mM of thiourea, 5 mM of sodium ascorbate, 3 mM of sodium pyruvate, 12 mM of *N*-acetylcysteine, adjusted to pH 7.3 and an osmolarity of 300–310 mOsmol, saturated with 95% O_2_/5% CO_2_) until use. Loose-Patch recordings from Purkinje cells were performed at room temperature in aCSF3 (containing: 125 mM of NaCl, 25 mM of NaHCO_3_, 25 mM of glucose, 2.5 mM of KCl, 1.25 mM of NaH_2_PO_4_, 1 mM of MgCl_2_, 2 mM of CaCl_2_, saturated with 95% O_2_/5% CO_2_), using thick-walled borosilicate glass recording electrodes (2.0 mm o.d., Science Products, Germany) filled with aCSF3 and a final resistance of 2–3 MOhm. Spontaneous tonic Purkinje cell activity was analyzed over a period of 5 s. Evoked responses were elicited by stimulating parallel fibers about 200 μm away from the Purkinje cell bodies using a monopolar stimulation with an additional pipette containing aCSF3 (DS3, Digitimer). Frequency and interspike interval were analyzed with the Neuromatic Plugin of IgorPro software (WaveMetrics, Portland, OR, United States).

### Statistical Analysis

Statistical analyses were performed using GraphPad Prism 8, Sigmaplot 13, and MATLAB 2020. First, the data sets were subjected to the Shapiro–Wilk test to check for the normal distribution. If the data sets passed the Shapiro–Wilk test (*p* value > 0.05), Student’s *t*-test was used to determine the statistical significance. If the data set did not pass the Shapiro–Wilk test (*p* value < 0.05), the statistical significance was determined by Mann–Whitney *U* test. One-way ANOVA was used for the comparison of more than one group. The exact *p* values are provided unless it is <0.001, and *p* < 0.05 was accepted as the level of significance for all of the tests. Methods that are more detailed in statistical analysis were described in the figure legends.

## Results

### Deletion of *Nbs1* in Purkinje Cells Disrupts the MRN Complex but Is Compatible With Mouse Development

**FIGURE 1:**
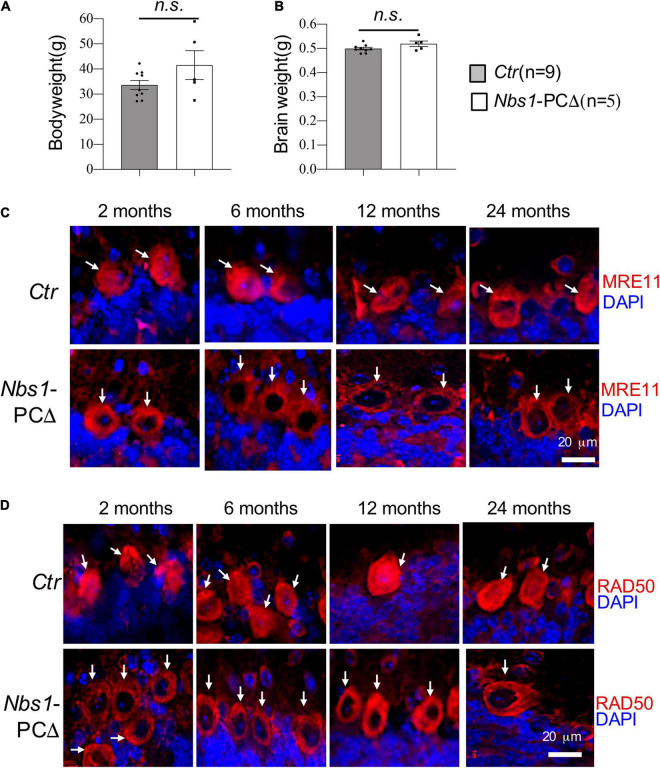
Deletion of *Nbs1* is compatible with mouse survival and cerebellar development. **(A)** Bodyweight of 20∼24 months old mice from the indicated group. **(B)** Brain weight of the animal for panel **(A)**, Error bars indicate the value mean ± SEM; n.s., not significant. Data comparison was performed through Student’s *t*-test. **(C,D)** Deletion of *Nbs1* in Purkinje cells leads to dislocation of MRE11 and RAD50. Sagittal cryo-sections of control (*Ctr*) and *Nbs1*-PCΔ mice at the indicated age were stained with anti-MRE11 **(C)** and anti-RAD50 **(D)** antibodies together with DAPI to label nuclei. The Purkinje cell layer in the cerebellum region is shown. Scale bars: 20 μm. Representative images of sections from two mice are shown. Arrows point to Purkinje cells.

To investigate the function of NBS1 in postmitotic neurons-Purkinje cells (PC), we generated *Nbs1*-PCΔ by crossing *Nbs1^flox^* mice (Frappart et al., 2005) with L7/pcp2-Cre transgenic mice (Barski et al., 2000), which allows specific deletion of *Nbs1* in Purkinje cells in the cerebellum. *Nbs1*-PCΔ mice were born normally and showed no obvious phenotype during the period of 2.5 years. The body weight and brain weight are similar between *Nbs1*-PCΔ and controls (*Ctr*) at all ages (Figures 1A,B, showed data from 2 years old).

To confirm the deletion of NBS1 in Purkinje cells, immunostaining with antibodies against NBS1 was performed. Unfortunately, none of the tested-commercial and -homemade antibodies worked specifically to detect NBS1 (data not shown). It has been shown that deletion of NBS1 impairs the nuclear localization of other components of the MRN complex in cells (Carney et al., 1998; Bruhn et al., 2014; Zhou et al., 2020). We next stained the cerebellar sections firstly from 2 months old animals with MRE11 antibodies. In contrast to controls which showed a robust MRE11 antibody reactivity in the Purkinje cells, both in the nucleus and cytosol (Figure 1C), the nuclear localization of MRE11 is nearly absent in the Purkinje cells from *Nbs1*-PCΔ animals (Figure 1C). The delocalization of MRE11 from the nucleus to the cytoplasm, i.e., the absence of the nuclear staining of MRE11, was observed throughout all ages in an observation period of 24 months (Figure 1C). To further confirm the MRN complex’s deficiency in *Nbs1*-deleted Purkinje cells, an anti-RAD50 antibody was also applied for immunostaining, which showed dislocation of RAD50 signals again from the nucleus to the cytosol of Purkinje cells (Figure 1D). All these observations strongly suggest that the NBS1 deletion specifically disrupted the MRN complex in the Purkinje cells.

### DNA Damage Response Is Impaired in *Nbs1*-PCΔ Purkinje Cells

**FIGURE 2:**
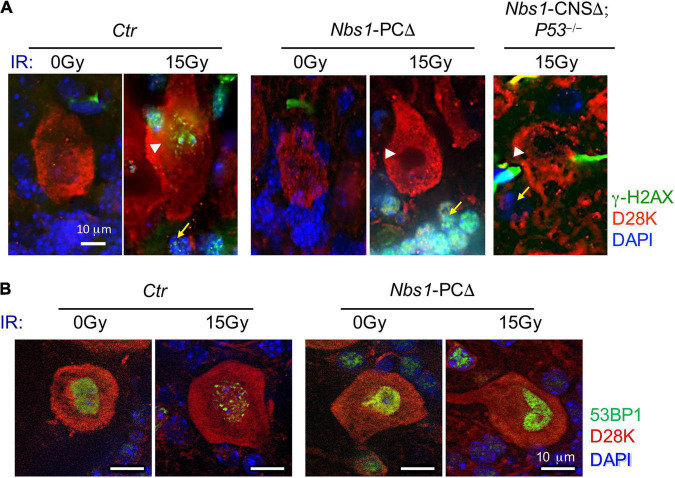
DDR was impaired in *Nbs1*-deleted Purkinje cells. **(A)** Analysis of DNA damage response. Immunostaining of Purkinje cells using γ-H2AX antibody on sections of 2-month-old control (*Ctr*), *Nbs1* Purkinje cells -deleted (*Nbs1*-PCΔ) as well as *Nbs1*-CNSΔ; *P53*^– /–^ mice without (0 Gy) or with 15 Gy ionizing radiation (IR). The sagittal cryosections 30 min post-IR were stained with DAPI (blue), Calbindin-D28K (D28K, red), and γ-H2AX (green) to label DNA, Purkinje cells, and DNA damage foci, respectively. The Purkinje cell layer is shown. Arrowheads point to Purkinje cells, arrows point to granule cells. *Nbs1*-CNSΔ; *P53*^– /–^ brain section is used as a negative control of DNA damage response for *Nbs1* knockout granular cells. **(B)** *Ctr* or *Nbs1*-PCΔ brain section with (15 Gy) or without (0 Gy) IR from panel **(A)** were stained with DAPI (blue), Calbindin-D28K (D28K, red), and 53BP1 (green) to label DNA, Purkinje cells, and earl DNA damage foci, respectively. Scale bars: 10 μm. Representative images of sections from two mice are shown.

Since we found a prominent expression of MRE11 or RAD50 in wild-type Purkinje cells (Figures 1C,D) and dislocation of MRE11 and RAD50 in *Nbs1*-PCΔ mice, we next tested the DDR singling in *Nbs1*-deleted Purkinje cells after acute DNA damage by 15 Gy of ionizing radiation (IR). Immunofluorescent staining of cerebella tissues revealed intense γ-H2AX foci signals in both Purkinje cells (white arrowhead) and their surrounding granule cells (yellow arrow) on the section from irradiated, but not the untreated, wild type animals (*Ctr*) (Figure 2A). Strikingly, γ-H2AX foci were utterly absent in *Nbs1*-deleted Purkinje cells after IR, whereas the γ-H2AX foci were strongly induced in the surrounding granule cells (yellow arrow) (Figure 2A). To further confirm NBS1 is required for IR-induced γ-H2AX foci in Purkinje cells and granule cells, we analyzed γ-H2AX focus formation in *Nbs1*-CNSΔ; *P*53^–/–^ animals in which *Nbs1* is deleted in both Purkinje cells and granule cells (Frappart et al., 2005). Interestingly, although deletion of *P53*, a key executor of DDR, partially rescues the Ataxia phenotype in *Nbs1*-CNSΔ (Frappart et al., 2005), γ-H2AX foci formation was abolished in Purkinje cells and surrounding granule cells in the absence of NBS1 (Figure 2A). These results demonstrate that proper DNA damage signaling, evidenced in *Nbs1* non-deleted surrounding granule cells, is lacking in Purkinje cells once the MRN complex is impaired. We next monitored the focus formation of the early DDR molecule 53BP1 in *Nbs1*-deleted Purkinje cells. As shown in Figure 2B, the 53BP1 focus formation was attenuated in Purkinje cells from IR-treated *Nbs1*-PCΔ compared to the irradiated-control cerebellar tissues (*Ctr*) (Figure 2B), indicating a defective DDR signaling in *Nbs1*-null Purkinje cells. Taken all together, these data indicate that deletion of NBS1 disrupts the DDR function of the MRN complex, which is required for proper DDR signaling in Purkinje cells.

### Normal Cerebellar Development and Purkinje Cells Are Associated With Regular Locomotor Function in *Nbs1*-PCΔ Mice

**FIGURE 3:**
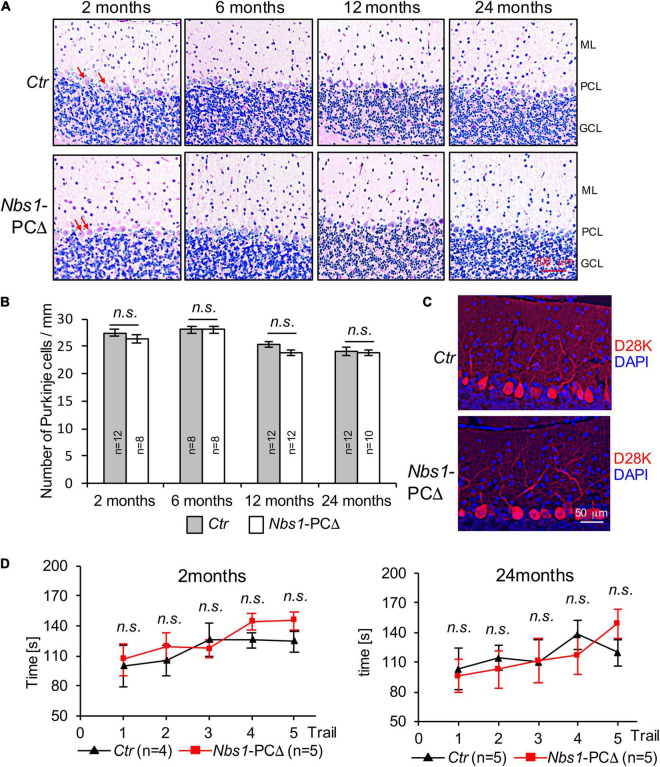
*Nbs1*-PCΔ mice show normal cerebellar morphology and the density of Purkinje cells. **(A)** Hematoxylin and eosin staining (H&E) of sagittal cerebellar sections of control (*Ctr*) and *Nbs1*-PCΔ mouse of the indicated age. The middle region of the lobule V of the cerebellum is shown. ML: molecular layer, PCL: Purkinje cell layer, GCL: granular cell layer. Arrows point to Purkinje cells. **(B)** Quantification of Purkinje cell number of the cerebellum of the control (*Ctr*) and the *Nbs1*-PCΔ mouse at the indicated age. The data is represented as the number of Purkinje cells per micrometer of the lobule V. The number of mice is indicated within the bar. The Student’s *t*-test is performed for the statistical analysis between control and *Nbs1*-PCΔ. n.s., not significant. **(C)** Morphology of Purkinje cells was analyzed by immunostaining with Calbindin-D28K (D28K) for Purkinje cells and DAPI for the nucleus of 2-month-old control (*Ctr*) and *Nbs1*-PCΔ mouse brain sagittal sections. Representative images of the middle region of the lobule V of the cerebellum from two mice are shown. **(D)** *Nbs1*-PCΔ mice have the normal locomotor capability. The rotarod performance of 2-month-old (left) and 24-month-old (right) mice on five consecutive days with the indicated number of control (*Ctr*) and *Nbs1*-PCΔ mice. The Student’s *t*-test is performed for the statistical analysis between control and *Nbs1*-PCΔ at each trial. n.s., no significant.

To study the effect of NBS1 deletion in the development and survival of Purkinje cells and cerebella, we performed histological analyses. The quantification revealed that the density of Purkinje cells was similar between control (*Ctr*) and *Nbs1*-PCΔ mice at a young age (2 months) (Figures 3A,B). Although the density of Purkinje cells was gradually reduced during aging, deletion of *Nbs1* did not accelerate this process (Figures 3A,B). *Nbs1*-PCΔ cerebella had a similar density of Purkinje cells, even at the age of 24 months, compared to control animals (Figure 3B). Immunofluorescence staining on the cerebellar sections with the Purkinje cell marker Calbindin-D28K (D28K) showed that *Nbs1*-deleted Purkinje cells had a similar morphology pattern as control littermates (Figure 3C), suggesting that knockout of *Nbs1* specifically in Purkinje cells has no effect on the morphogenesis and survival of Purkinje cells.

Consistent with the observation of a normal density and morphology of Purkinje cells, *Nbs1*-PCΔ mice, at a young age (2-month), spent similar time on the accelerated Rotarod as controls (Figure 3D, left). Moreover, even at the age of 2 years, no significant difference was found between *Nbs1*-PCΔ mice and the littermate controls in the Rotarod tests (Figure 3D, right). These results indicate that *Nbs1* deletion in Purkinje cells has a negligible effect on the locomotor coordination capability regardless of age. Taken together, these results indicate that the deletion of NBS1, which disrupted the MRN complex-mediated DDR signaling, does not compromise the survival and function of Purkinje cells during cerebellar development and aging.

### Specific Deletion of *Mre11* in Purkinje Cells Is Compatible With the Development and Survival of Animals

**FIGURE 4:**
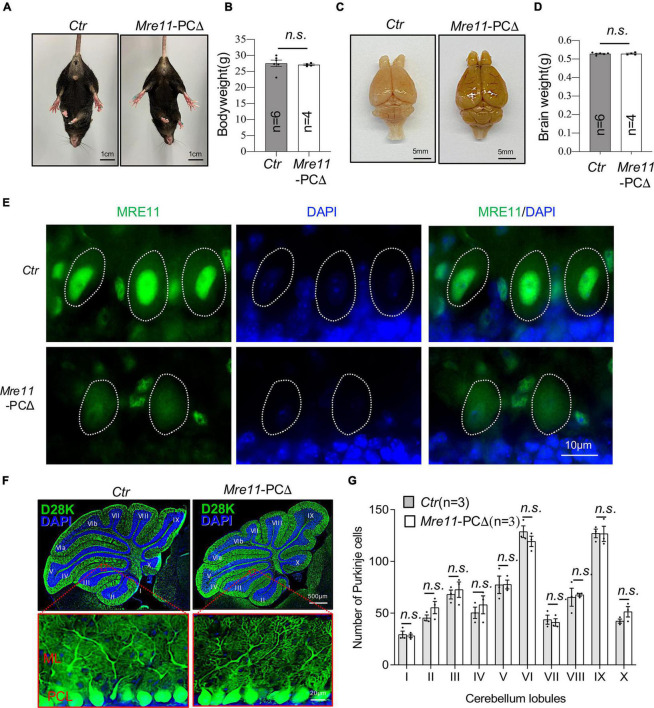
Normal brain and the cerebellum of *Mre11*-PCΔ mice. **(A)** Abdominal view of wild type (*Ctr*) and *Mre11*-PCΔ mice showed normal limbs stretching. **(B)** Bodyweight of control (*Ctr*) and *Mre11*-PCΔ mice at 18 months of age. The number of mice analyzed is indicated within the graph bars. Error bars indicate the value mean ± SEM; n.s., not significant; data comparison was performed through Student’s *t*-test. **(C)** A dorsal view of the whole brain of control (*Ctr*) and *Mre11*-PCΔ mice. **(D)** Brain weight of 12-month-old *Ctr* and *Mre11*-PCΔ mice. The number of mice analyzed is indicated within the graph bars. Error bars indicate the value mean ± SEM; data comparison was performed by Student’s *t*-test. n.s., not significant. **(E)** Sagittal cryosections of 18-month-old *Ctr* and *Mre11*-PCΔ mice after staining with anti-MRE11 antibody and DAPI. Note a reduction of MRE11 signal in mutant Purkinje cells. The Purkinje cell layer in the cerebellum region is shown. Dotted lines highlight the nucleus of Purkinje cells. **(F,G)** *Mre11*-PCΔ mice have normal density and morphology of Purkinje cells. Sagittal sections of control (*Ctr*) and *Mre11*-PCΔ mice were stained with Calbindin-D28K (D28K) antibody (green) and DAPI (blue) to label Purkinje cells and nuclei, respectively **(F)**. The upper panels show the whole cerebellum, and the lower panels show the magnified view of red frames in the respective upper panels. ML, molecular layer; PCL, Purkinje cell layer. **(G)** Quantification of total Purkinje cell number in each individual cerebellar lobule from panel **(F)**. Three mice of each genotype were analyzed. Error bars indicate the value mean ± SEM; data comparison was performed through Student’s *t*-test. n.s., not significant.

MRE11 mutations cause the A-TLD syndrome, which, by its definition, shows cerebellar defects and ataxia (Stracker and Petrini, 2011). To further dissect the functional difference of each component of the MRN complex in Purkinje cells, we generated another mouse model (*Mre11*-PCΔ), in which *Mre11* was deleted in Purkinje cells by crossing *Mre11 *^flox^** mice (Buis et al., 2008) with *L7/pcp2*-Cre transgenic mice (Barski et al., 2000). These mutant mice were born normally and showed no obvious phenotype up to 24 months (Figure 4A, data not shown). Neither the bodyweight nor the brain weight of *Mre11*-PCΔ mice had any difference compared to control littermates (Figures 4B–D). The mutant mice showed regular locomotor coordination based on the tail lifting monitoring assay and the walking behavior test (Figure 4A, data not shown). Immunofluorescence staining of cerebellar sections revealed a dramatic decrease of MRE11 signal in mutant Purkinje cells, indicating an efficient deletion of MRE11 (Figure 4E). These data indicate that MRE11 is dispensable for the development and survival of mice if it is deleted in postmitotic Purkinje cells.

Immunofluorescence staining of the cerebellar section by Calbindin-D28K (D28K) revealed a similar morphology of Purkinje cells between *Mre11*-PCΔ mice and littermate controls (Figure 4F, low panel). Moreover, the densities of Purkinje cells in all lobules of the cerebellum were similar between *Mre11*-PCΔ and control animals at the age of 18 months (Figure 4G). All these results indicate that MRN plays an ignorable role in maintaining the morphology and the survival of Purkinje cells.

### Deletion of *Mre11* Does Not Lead to Accumulation of DNA Damage in Purkinje Cells

**FIGURE 5:**
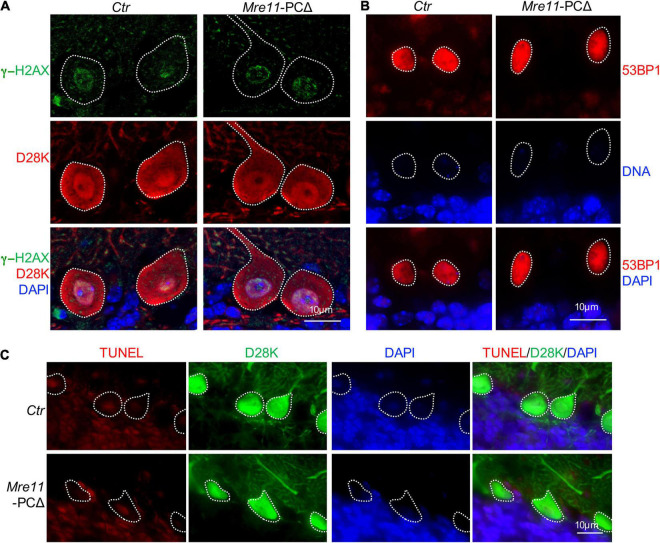
Deletion of *Mre11* did not lead to damage accumulation in Purkinje cells. **(A)** Sagittal sections of 18-month-old control (*Ctr*) and *Mre11*-PCΔ mice were stained with γ-H2AX (green), Calbindin-D28K (D28K, red), and DAPI (blue) to label DNA damage, Purkinje cells, and DNA, respectively. Dotted lines highlight Purkinje cells. **(B)** Sagittal sections of 18-month-old *Ctr* and *Mre11*-PCΔ mice were stained with 53BP1 (red) and DAPI (blue) to label damaged DNA and nuclei, respectively. Dotted lines highlight the nucleus of Purkinje cells. **(C)** Sagittal sections of 18-month-old *Ctr* and *Mre11*-PCΔ mice were stained with Calbindin-D28K (D28K, green), TUNEL (red), and DAPI (blue) to label Purkinje cells, apoptotic cells, and DNA, respectively. Dotted lines highlight Purkinje cells.

MRE11 functions to repair replication-associated DNA double-strand breaks (DSBs), and deletion of *Mre11* leads to accumulation of damaged DNA and cell death in proliferating cells (Costanzo et al., 2001). To examine whether deletion of *Mre11* in Purkinje cells also causes DNA damage accumulation, the cerebellar sections were first stained with an antibody against γ-H2AX, which did not detect clear foci in Purkinje cells from both *Mre11*-PCΔ and control animals (Figure 5A). To further confirm whether there is an accumulation of DNA damage, we stained the cerebellar sections with an antibody against the early DDR molecule 53BP1. 53BP1 signal was found in a diffused pattern, without any detectable foci, in the nucleus of Purkinje cells from both *Mre11*- PCΔ and control (*Ctr*) mice under unperturbed conditions (Figure 5B). Moreover, TUNEL staining did not detect any apparent cell death in *Mre11*-PCΔ Purkinje cells (Figure 5C). All these data suggest that deletion of *Mre11* caused neither accumulation of damaged DNA nor cell death in Purkinje cells.

### MRE11 Is Dispensable for the Intrinsic Activity of Purkinje Cells

**FIGURE 6:**
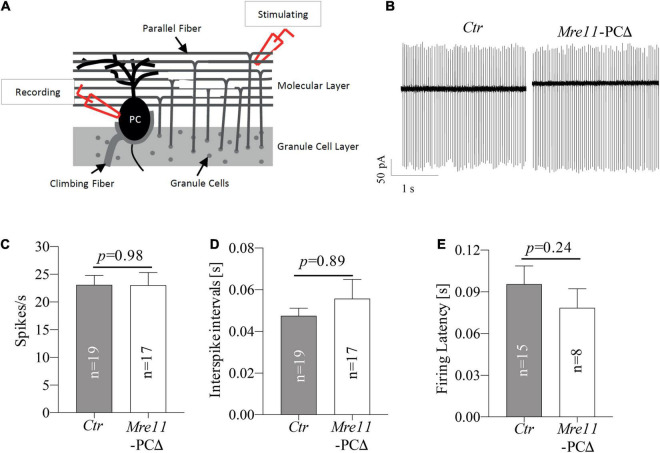
*Mre11*-PCΔ Purkinje cells have regular intrinsic activity. **(A)** Schematic representation of cerebellar loose-patch electrophysiology measurements on Purkinje cell soma and stimulation of parallel fiber-Purkinje cell synapses. PC: Purkinje cell. **(B)** Sample traces from passive recordings of the spontaneous tonic activity of Purkinje cells in controls (*Ctr*) and *Mre11*-PCΔ mice at the age of 18 months. **(C)** Passive tonic activity as shown by spikes per second (spikes/s) of control (*Ctr*) and *Mre11*-PCΔ neurons. **(D)** Interspike intervals (in s). **(E)** The firing latency is measured as the time from the parallel fiber stimulation evoked Purkinje cell spike to the next spike of Purkinje cells (in s). The number of neurons measured is indicated within the graph. The *P*-value of the Student’s *t*-test is shown in panels **(C–E)**.

The Purkinje cells control locomotor function by conducting the main output of the inhibitory circuit. We investigated the impact of *Mre11* deletion on the neuronal activity of Purkinje cells. Loose-patch electrophysiological recordings on Purkinje cells of 18 months old mice were carried out to evaluate the intrinsic activity (Figure 6A). We found that both the spontaneous tonic spiking frequency and interspike intervals (ISI) of Purkinje cells were comparable between *Mre11*-PCΔ mice and controls (*Ctr*) (Figures 6B–D). We further monitored the parallel fiber-evoked (PF) activity and measured the firing latency of Purkinje cells following the first evoked spike after PF stimulation. *Mre11*-deleted Purkinje cells responded to PF stimulation similarly to controls, judged by a similar spiking delay as controls (Figure 6E). Taken together, MRE11 does not seem to play a significant role in regulating the intrinsic activity of Purkinje cells.

## Discussion

Human genomic instability syndromes, caused by deficiency of the MRN complex, are characterized by neurological deficits, including microcephaly and ataxia (McKinnon, 2009, 2017; Kanner et al., 2018). Whereas primary microcephaly is generally caused by the loss of proliferation and survival of embryonic neuroprogenitors (Jayaraman et al., 2018), ataxia is due to the block of cerebellar development that is thought to be the consequence of the cerebellar defect and dysfunction or degeneration of Purkinje cells (Hoxha et al., 2018). Purkinje cells have an extensive transcriptional activity that keeps large portions of their chromatin in de-condensed conditions (Tzur-Gilat et al., 2013) and, additionally, also harbor a high level of reactive oxygen species (ROS), which can induce DNA damage (Madabhushi et al., 2014). Therefore, Purkinje cells are proposed to be vulnerable to malformation of DDR. Indeed, progressive cerebellar degeneration, accompanied by the loss of Purkinje cells, has been reported in A-T, A-TLD, and other genomic instability syndromes (Taylor et al., 2004; Jeppesen et al., 2011; Madabhushi et al., 2014). For instance, mutations of SSB repair gene APTX lead to oculomotor apraxia-1 (AOA1), in which cerebellar atrophy caused by a severe loss of Purkinje cells are characterized along with oculomotor apraxia (Date et al., 2001; Moreira et al., 2001). Here we show, by specific deletion of *Nbs1* or *Mre11* in Purkinje cells, that the MRN complex plays a neglectable role in maintaining the cerebellum and the survival as well as the activity of Purkinje cells in mouse models (see Figures 3, 4, 6). Consist with the “non-essential” function of MRE11 and NBS1 in postmitotic neuron Purkinje cells, although expression of the “essential” kinase-dead ATM (ATM^KD^) leads to embryonic lethality, conditional expression of ATM^KD^ in mouse cerebellum did not cause any Purkinje cell loss or significant neurological phenotype (Tal et al., 2018). Moreover, depletion of *Rad50* in postmitotic hepatocytes caused neither cell death in the liver nor any other obvious phenotype in a mouse model (Adelman et al., 2009).

The observations that deletion of *Nbs1* or *Mre11* in Purkinje cells do not phenocopy cerebellar degeneration and ataxia in human A-TLD patients are surprising. The possibility could be that cerebellar degeneration and ataxia in human patients is an outcome of dysregulation of progenitors of Purkinje cells due to the absence of DDR function after mutations of these genes in germ cells. Indeed, severe cerebellar atrophy and ataxia were found in *Nestin*-Cre mediated deletion of *Nbs1* in progenitors of CNS (Frappart et al., 2005). Mouse progenitors of Purkinje cells appear during early fetal development (embryonic days 11–13 in mice), migrate to the cerebellar rudiment, and undergo dramatic expansion only at birth and early postnatal life, maturation of Purkinje cells takes place in 2∼3 weeks after birth (Dusart and Flamant, 2012). In the current study, the deletion of *Nbs1* or *Mre11* mediated by L7/pcp2-Cre only starts from postnatal day 7 (P7) when the Purkinje cells are already formed (Barski et al., 2000; Dusart and Flamant, 2012). Therefore, the defect of cerebellum development and survival of Purkinje cells in *Nbs1*-CNSΔ mouse model could be a consequence of the malfunction of progenitors of Purkinje cells. Moreover, it is interesting to note that deletions of *Atr* in mouse neuroprogenitors (mediated by *Nestin*-Cre) also caused cerebellar atrophy and ataxia, although these phenotypes were absent in the Seckel Syndrome (ATR-SS). Unlike its role in proliferating progenitors (Frappart et al., 2005), the MRN complex is not essential for the maturation and survival of postmitotic Purkinje cells. Although impairment of DDR response (tested by γ-H2AX and 53BP1 focus formation after IR treatment) was evidenced in *Nbs1*-PCΔ cerebellar Purkinje cells (Figure 2), no noticeable accumulation of DNA damage was detectable in mutant Purkinje cells under physiological conditions (Figures 2, 5), which could be due to the fact that vastly different lifespan between the two species that may have a significant impact on their response strategies to deal with DNA damage, in which the relatively short life expectancy of mice might preclude the appearance of effects of DDR-deficiency that manifest over two decades in human Purkinje cells (Madabhushi et al., 2014). Nevertheless, considering severe cerebellar atrophy and ataxia in mouse models with deletion of the *Nbs1* or *Atr* in the neuroprogenitors (*Nbs1*-CNSΔ or *Atr*-CNSΔ) and the absence of cerebellar deficits of both *Nbs1*-PCΔ and *Mre11*-PCΔ mice, neuronal degeneration found in A-TLD (even A-T) patients (Jackson and Bartek, 2009; Chrzanowska et al., 2012), are most likely of a developmental origin of the impaired function of the MRN complex rather than an intrinsic function of this complex in postmitotic Purkinje cells (Stracker and Petrini, 2011; Lee et al., 2012). Consistent with this, deletion of the *Mre11* in the neuroprogenitors (*Mre11*-CNSΔ) leads to severe cerebellar atrophy and ataxia (Qing et al., unpublished).

Another possibility that causes cerebellar atrophy and ataxia in *Nbs1*- CNSΔ, *Mre11*-CNSΔ, or *Atr*- CNSΔ could be due to the impact of DDR defect in surrounding cells of Purkinje cells or neurons. Indeed, an abolished or impaired expression of *Nbs1* was found in astrocytes or microglial cells in *Nbs1*-CNSΔ animals, which showed a malfunctioning astrocyte activity (Dar et al., 2011), indicating a possible impact on Purkinje cells by surrounding cells such as astrocyte, microglia. More interestingly, a recent study shows that wild-type astrocytes can restore connectivity and synchronization in the ATM-deleted neuronal networks (Kanner et al., 2018). All of these strongly support the possibility that the atrophy and ataxia in A-T, A-TLD patients may be due to the major impact on Purkinje cells through surrounding cells. For that, it is fascinating to create mouse models with deletion of the MRN complex or expression ATM^KD^ in astrocyte or microglia cells and investigate the ataxia or neurodegeneration phenotype in them.

Nevertheless, the MRN complex, at least MRE11 or RAD50, is highly expressed in Purkinje cells (see Figures 1C,D, 4E), suggesting unknown physiological functions of these essential DDR molecules in these cells. Although the MRN complex is dispensable for the survival of postmitotic Purkinje cells, when *Nbs1* was deleted in differentiating neurons, the arborization and migration of neurons are impaired *in vitro* and *in vivo* (Zhou et al., 2020). Interestingly, silencing *Mre11* or *Rad50* resulted in different neuronal phenotypes compared to *Nbs1* mutants: knockdown of *Mre11* inhibits both the initiation and migration of newborn neurons in contrast to knockdown of *Nbs1* that only blocked the migration process. Surprisingly, *Rad50* deletion affects neither survival nor migration (Zhou et al., 2020). Molecular analyses demonstrated that these selective specificities of each component of the MRN complex could be due to their different interaction function to Notch signaling in regulating neuronal development and migration process (Zhou et al., 2020). Therefore, although the survival of postmitotic neuronal cells is not affected, differentiating neurons respond differently to the deletion of different components of the MRN complex, which could be due to interrupt off a non-canonical function of each component of the MRN complex during the differentiation process and therefore results in different symptoms in MRN-deficiency associated patients (Jackson and Bartek, 2009; Chrzanowska et al., 2012).

Furthermore, although the MRN complex and ATR are all essential DDR molecules and the functions of the MRN complex also on upstream of ATR in the DDR pathway depending on cell cycle status and physiological status, deletion of *Atr* in Purkinje cells (*Atr*-PCΔ mice) does not compromise the development and survival of Purkinje cells. However, *Atr*-PCΔ mice show ataxia due to a defect of intrinsic neuronal activity mediated by modulating presynaptic firing through interacting between ATR and synaptotagmin 2 (SYT2), rather than cerebellar degeneration (Kirtay et al., 2021). In this regard, it is interesting to note that Purkinje cells from *Mre11*-deleted mice do not show any electrophysiological defects in the current study (Figure 6). Therefore, the “non-canonical” functions that may respond to the different symptoms in MRN-deficient patients are required for further investigation.

Taken together of the current study and previously published work, we conclude that apart from their critical DDR functions in proliferation and cell death, the essential MRN complex and ATR play a “non-canonical DDR function” in specific a population of non-proliferating cells, such as Purkinje neurons. These studies further suggest the physiological function of these factors in etiologies of the corresponding genomic instability syndromes beyond their DDR function.

## Data Availability Statement

The raw data supporting the conclusions of this article will be made available by the authors, without undue reservation.

## Ethics Statement

The animal study was reviewed and approved by (1) Thüringer Landesverwaltungsamt (animal license 03-042/16), Germany; (2) SYSU Institutional Animal Care and Use Committee (SYSU-IACUC-2019-B1028), China. Written informed consent was obtained from the owners for the participation of their animals in this study.

## Author Contributions

MD performed most experiments, interpreted the data, and wrote the manuscript. XQ and GZ analyzed the number and survival of Purkinje cells from *Mre11*-PCΔ mice. RG and HL performed the experiments including behavior test and fluorescent staining relative to *Nbs1*-PCΔ. CB-B performed electrophysiological analyses. DF provided *Mre11* floxed mutant mice. CG supervised electrophysiological analyses. Z-QW conceived and wrote the manuscript. Z-WZ conceived and supervised the project and wrote the manuscript. All authors contributed to the article and approved the submitted version.

## Conflict of Interest

The authors declare that the research was conducted in the absence of any commercial or financial relationships that could be construed as a potential conflict of interest.

## Publisher’s Note

All claims expressed in this article are solely those of the authors and do not necessarily represent those of their affiliated organizations, or those of the publisher, the editors and the reviewers. Any product that may be evaluated in this article, or claim that may be made by its manufacturer, is not guaranteed or endorsed by the publisher.
